# Political Economy and Research Silos: A Response to the Recent Commentaries

**DOI:** 10.34172/ijhpm.2023.7995

**Published:** 2023-04-10

**Authors:** Jennifer Lacy-Nichols, Owain Williams

**Affiliations:** ^1^Centre for Health Policy, Melbourne School of Population and Global Health, University of Melbourne, Melbourne, VIC, Australia; ^2^School of Politics and International Studies, University of Leeds, Leeds, UK

## Introduction

 The responses to our paper “‘*Part of the Solution’: Food Corporation Strategies for Regulatory Capture and Legitimacy”*^1^ greatly expand on our case study of a shift in the strategy of powerful food companies and develop a myriad of new avenues for analysis. The strongest theme in the responses was the emphasis on how, practically, to counter the power and influence of commercial actors. A second theme considered how to go beyond a narrow focus on specific industry sectors and identify cross-cutting commonalities across commercial actors. A final thread challenged our assertion that there are dangers in working with industry actors and reflected on what trade-offs might be acceptable in the pursuit of public health. Here, we reflect first on our paper’s limitations. We then discuss the opportunities and challenges our colleagues’ proposed new avenues and agendas raise for our collective research around corporate and commercial determinants of health.

## Partial Political Economy

 The global political economy of food and health is complex and there are multiple issues by which to engage with the actions of food corporations. Our paper is only a part of that political economy of food and only one entry point into the role of powerful financial capital, corporate actors, markets and institutions in determining how and what we eat, where food is produced and the benefits that accrue from its commodification in various ways. Below, we interrogate our own narrow focus and spell out what we have missed that is of importance. We then make more explicit the underlying contexts in our original analysis.

 In the narrow sense, our paper mainly focused on regulatory and institutional dynamics such as being at the table, the revolving door, lobbying, political donations, rhetorical framing and forum shifting. In the language of industrial economics this is commonly termed firm *non-market strategy*. Corporate political power is crucial to understanding how big food preserves markets and influences politics, but it is clearly only one part of the political economy of corporate food. We suggest that, like much of the work in political economy since the 1980s, public health scholarship has taken its eye off what corporations are doing in terms of *market strategy *and standard business practices, such as sector concentration, mergers and acquisitions, product diversification, horizontal and vertical integration, value chains and financialization. These activities give a narrowing band food oligopolists extraordinary market position over a range of sectors concerned with agriculture and food and grant them power in structuring the markets in which we consume food.^2^ Without market power there would be less corporate political power. In the narrow sense, our political economy of food is much like the weight of scholarship – too focused on institutional and regulatory elements, therefore failing to capture the full picture of corporate strategy. We need to be more material and holistic in how we look at corporate power.

 Fundamentally, a political economy approach is about power and history – in this case, how the food system is a product of historical forces and social relations of production. So in the wider sense, our political economy is only one snapshot of a time-bound strategic response by corporations to a (potentially) hostile regulatory environment. What we also fail to articulate is what is often absent from political economies of food and public health, namely the need for a historical lens. The arc from colonialism, plantation systems and settler agriculture propelled the development of the early globalization food system. This process involved both slavery and wage slavery, mass expropriation of plant genetic resources from the Global South, and steady intensification of agriculture and commodity production and consumption. Crucially, the process involved commodification and enclosure, the granting of property rights for land and the exclusion of many from the commons and therefore the basic means of independence and sustainability in the context of increasingly commodified food.^3^ As Kloppenburg showed us, more industrialised means of food production were grounded in continued extraction, both in terms of labour and plant genetic resources. With intensifying commercial presence new sets of legal rights were established to ensure further commodification, as with the provision of plant breeders rights in the national systems of high-income countries, and later globalising systems of patents. Corporations have overlaid their presence on empire-constructed and industrialised food, albeit with increasingly sophisticated ownership structures, complex financialization and conglomeration.

 The same concentration in the commercial seed sector occurs in food retail and food processing. The names of the commercial entities are blurred by the logic of branding and rebranding after cross-border acquisitions and joint ventures. These market strategies are juggernaut and tie up almost all areas of the global food system, limiting alternatives and generating profits, whilst externalising harms to health, climate and the environment. Institutionally, these processes are tolerated (eg, a dilution of anti-trust) and co-produced by regulatory capture by big food. Transnational regulatory capture amplifies and globalises these processes, via proliferating trade agreements, the World Trade Organization, the Food and Agriculture Organization and new public-private food governance partnerships and philanthropies.

 In the present, there seems to a be an underlying and rapidly crystallising global political economy to the food system that we would hold is structurally pathogenic for the environment, ecosystems, climate change and human health.^4^ There is a palpable sense, evident in mobilisation around the United Nations Food Summit and COP 26 in 2021 that we have reached a point of inflection in human history, in food and in health, with the COVID-19 pandemic exposing the weaknesses in multiple global systems which we depend upon that have been generated by neoliberal capitalism.^5^ Thus, there is much more to do in constructing new political economies of food.

## Countering Vested Interests in Public Health

 Our colleagues offered many suggestions to expand our analysis. Freudenberg^6^ and Crosbie and Carriedo^7^ both document a series of counter strategies to challenge the dominance of vested interests in the food system. Crosbie and Carriedo explore several strategies to better understand and expose commercial strategies. They note that one of the key learnings from tobacco is the value of a deep dive, as it is the specific details that foster outrage and generate the enabling environments for regulators to act. This supports Freudenberg’s denormalization strategy, which hinges on exposing harmful and disingenuous practices. One challenge facing researchers and activists working to monitor and expose harmful commercial practices is the paradox of the sheer breadth of evidence and examples, while at the same time facing poor transparency of many practices. The true breadth of lobbying is rarely made public by governments, and when it is the data is disorganised and difficult to make sense of,^8^ or comes with restricted access.

 Baum and Anaf ^9^ ask what steps might be necessary to move beyond the current status quo, characterised by transnational corporations (TNCs) dominating most industry sectors. This approach looks beyond the specific portfolio of a company and highlights the commonalities across TNCs and the circumstances that enable their continued dominance. It also highlights the importance of actions targeting the enabling context for corporate power (ie, capitalism, neoliberalism, etc). As Lencucha^10^ elaborates, the current political context views all industries as natural contributors to the economy – it is within these structures that corporations act. But, they need not promote the interests of TNCs at the expense of health. What if our policies supported alternative forms of business, such as cooperatives or public ownership? Indeed, there is a growing movement of remunicipalisation around the world, with local and state governments taking back ownership from the private sector, actions which have improved health equity and the bottom line.^11^

## Moving Outside Our Research Silos

 Wiist^12^ urges us to move outside our research silos (food, tobacco, etc) and recognise that the practices are not unique to one sector. Refocusing attention from specific products and industry sectors instead to the types of commercial entities opens the door for a wider transformative change to our economic system. This is certainly the direction that commercial determinants of health scholarship is headed, with a widening focus on diverse industry sectors not traditionally viewed as health harming.^13,14^ This wider focus also open the door for new alliances and partnerships with like-minded advocacy organisations such as anti-corruption organisations, human rights groups, climate change activists and others often take corporate power as a starting point.

 How, conceptually and practically, do we move beyond the well-researched ‘harmful products’? Even these, as Lencucha^10^ notes, are often ambiguous – with no company or industry wholly health harming. If we look to the other end of the spectrum to health promoting services, such as childcare, healthcare, education, utilities like water and energy, these are essential, yet many are privatised, financialized and extractive. How can we begin to measure and evaluate the net health impact of a company or sector?

 One challenge is where to draw the boundary around the commercial actor. As Allen^15^ notes, assessments could occur at the level of product, a portfolio, or a global company. We would take this one step further and question when and where supply/value chains comes into play, or the company’s legal firms, or banks and other financial services. Virtually every commercial actor that is part of the formal economy (and many too that are part of the informal economy) will have ties to other businesses, whether that be their bank, or auditor, or their real estate agent or their utility company and others. Few, if any companies would be wholly self-sufficient and isolated from other businesses. To what end, then should they be held accountable for their choice of utility provider and their fossil fuel footprint? To what extent are they responsible for a portion of their bank’s financial practices, or their auditor’s (potential) role in tax evasion? Is there an expiry on former, harmful industry relationships? How many years must past before a de-merger or divestment has sufficiently distanced two entities?

 These questions are especially challenging when trying to build guidelines and rules for managing conflicts of interest (a key strategy to counter commercial influence). While a black and white line usually exists for the tobacco industry (however inadequately enforced), few such lines exist for other sectors. As Allen argues, each company is ostensibly distinct (though corporate ownership structures may blur boundaries at times) and attempts to group companies together based on shared characteristics risk homogenizing actors with diverse interests and practices. The food industry (a term that we admittedly use too loosely in our paper) is immensely diverse, comprising smallholder farmers and multinationals, public and private enterprises, minimally- and ultra-processed foods, and organisations that lobby aggressively and those that have never engaged in politics. However, observing that the food industry is diverse is not too controversial a statement. But if we say that the tobacco industry, or alcohol or weapons or other industry is diverse, the implied shades of grey and wiggle room where regulation is concerned. This is uncomfortable. Commercial complexity presents philosophical and practical challenges for analysis and action. Determining when and why to compromise is challenging.

 It is impractical to treat each company as unique. Heuristics (ie, ways to simplify and make sense of things) are needed. The corporate health impact assessment framework developed by Baum and colleagues^16^ offers one such scheme to organise and make sense of information about TNCs. In his response, Wiist^12^ also notes the similarities in political practices across many industry sectors, and we agree that a much deeper understanding of corporate political and market strategy activity is necessary for public health and others to effectively counter commercial forces. This could include deep dives into industry archives and interviews with informants, as detailed by Crosbie and Carriedo.^7^ It must also include empirical analyses of corporate lobbying, an area of research often stymied by limited government disclosure. There is movement in this space, for instance the Organisation for Economic Co-operation and Development is currently updating its recommendations for lobbying transparency, and we look forward to thinking through how the public health community can support efforts to protect democracy and decrease corruption.^17^

## What Weight, Public Health?

 Allen^15^ asks the provocative question if public health ends justify trade-offs in other spaces. Respectfully, this is a brilliant question and impossible to answer. The question reminds us Crawford’s treatise on healthism, which he defines as “the preoccupation with health as a primary — often *the* primary — focus for the definition and achievement of well-being.”^18^ But should health always be prioritised, even at the expense of other values or ideals? And how (any by whom) should “health” be defined?

 In an ideal world, such trade-offs are not necessary. The world is far from ideal. To the question of whether increasing profits poses a risk to public health, we would argue yes, sort of. Irrespective of how profits are made, if already powerful companies become more powerful and better resourced, they are better equipped to influence governments and write the rules of the game. As Galbraith argued, this is a risk to democracy.^19^ For us, questions of democracy, power and control must be elevated over questions of health (which they ultimately determine). This is the crux of political economy.

## Ethical issues

 Not applicable.

## Competing interests

 Conceptualization: Jennifer Lacy-Nichols and Owain Williams.

 Writing–original draft: Jennifer Lacy-Nichols and Owain Williams.

 Writing–review & editing: Jennifer Lacy-Nichols and Owain Williams.

## Authors’ contributions

 JLN and OW developed the concept for the correspondence and both collaborated on the first draft of the correspondence and subsequent iterations. All authors read and approved the final manuscript.
